# An *In Vivo* Microdialysis Study of FLZ Penetration through the Blood-Brain Barrier in Normal and 6-Hydroxydopamine Induced Parkinson's Disease Model Rats

**DOI:** 10.1155/2014/850493

**Published:** 2014-06-23

**Authors:** Jinfeng Hou, Qian Liu, Yingfei Li, Hua Sun, Jinlan Zhang

**Affiliations:** State Key Laboratory of Bioactive Substance and Function of Natural Medicines, Institute of Materia Medica, Chinese Academy of Medical Sciences and Peking Union Medical College, Beijing 100050, China

## Abstract

FLZ (N-[2-(4-hydroxy-phenyl)-ethyl]-2-(2,5-dimethoxy-phenyl)-3-(3-methoxy-4-hydroxy-phenyl)-acrylamide) is a novel synthetic squamosamide derivative and a potential anti-Parkinson's disease (PD) agent. The objective of the present study was to investigate the penetration of free FLZ across the BBB and the effects of P-gp inhibition on FLZ transport in normal and 6-hydroxydopamine (6-OHDA) induced PD model rats. *In vivo* microdialysis was used to collect FLZ containing brain and blood dialysates following intravenous (i.v.) drug administration either with or without pretreatment with the specific P-gp inhibitor, zosuquidar trihydrochloride (zosuquidar*·*3HCl). A sensitive, rapid, and reliable ultraperformance liquid chromatography-tandem mass spectrometry (UPLC-MS/MS) technique was developed and validated to quantitate free FLZ levels in the dialysates. No significant differences were observed in the brain/blood FLZ area under the concentration-time curve (AUC) ratio between normal and PD model rats. However, pretreatment with zosuquidar*·*3HCl markedly increased the AUC ratio in both rat models. In addition, FLZ penetration was similar in zosuquidar*·*3HCl-pretreated normal and PD rats. These results suggest that P-gp inhibition increases BBB permeability to FLZ, thereby supporting the hypothesis that P-gp normally restricts FLZ transfer to the brain. These findings could provide reference data for future clinical trials and may aid investigation of the BBB permeability of other CNS-active substances.

## 1. Introduction

Parkinson's disease (PD) is the second most common neurodegenerative disorder after Alzheimer's disease (AD). The prevalence of PD increases with age and is approximately 0.3% in the general population, which rises rapidly to 3% for individuals over the age of 65 years [1].

Since the early 1960s, the standard treatment for PD has involved the pharmacologic replacement of dopamine with the dopamine precursor, 3,4-dihydroxy-L-phenylalanine (L-DOPA). Supplementation of L-DOPA with carbidopa, an inhibitor of aromatic L-amino acid decarboxylase (and hence the peripheral metabolism of L-DOPA), represented a marked improvement in therapy and is still a mainstay of PD treatment [2]. Many other pharmacotherapies, such as dopamine agonists, catechol-O-methyltransferase (COMT) inhibitors, monoamine oxidase-B (MAO-B) inhibitors, anticholinergic agents, amantadine, and glutamate receptor antagonists, have also been used [3]. However, there is still no cure for PD and no solid evidence for efficacious disease-modifying strategies. In addition, motor complications in the advanced stages of the disease, adverse effects of dopaminergic therapy, and nonmotor symptoms (e.g., loss of sense of smell, sleep disturbances, mood disorders, orthostatic hypotension, and constipation) remain enormous challenges during long-term therapy. Thus, new neuroprotective therapeutic agents are urgently needed [3].

**Figure 1:**
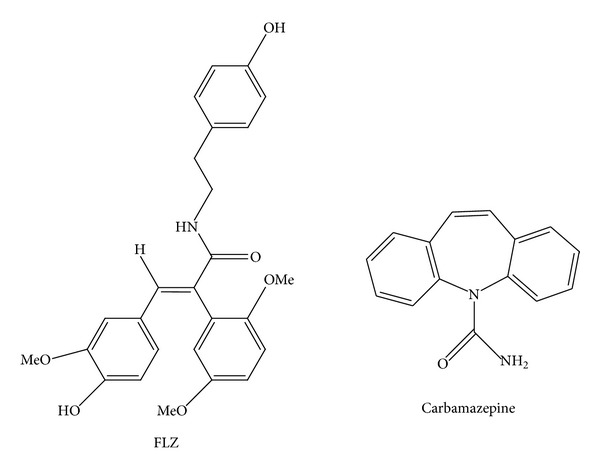
Structure of FLZ and carbamazepine (IS).

FLZ (N-[2-(4-hydroxy-phenyl)-ethyl]-2-(2,5-dimethoxy-phenyl)-3-(3-methoxy-4-hydroxy-phenyl)-acrylamide; Figure 1) is a novel synthetic derivative of squamosamide, which was first isolated from* Annona glabra *(Pond Apple) [4]. FLZ inhibits the lipopolysaccharide-induced production of certain inflammatory mediators [5], has potent neuroprotective effects [6–12], and may be a potential treatment for PD [6, 7, 13] and AD [8, 14–16].

A preclinical pharmacokinetic study showed that FLZ can cross the blood-brain barrier (BBB) with no target effects [17]. Our previous work also showed that the binding rate of FLZ to plasma proteins is very high (about 90%). Therefore, for therapeutic purposes, it is critical to examine the ability of free FLZ to cross the BBB. The BBB permeability of a drug can usually be established by sampling the cerebrospinal fluid (CSF) [18] or extracellular fluid (ECF) using a technique called microdialysis [19]. Microdialysis has a number of advantages over traditional methods [20–22]. For example, sampling can be performed dynamically and continuously with no fluid loss, and high-resolution concentration profiles for drugs and metabolites can be obtained from individual subjects. In addition, only drugs that penetrate the BBB are obtained using this technique, whereas samples obtained from brain homogenates also contain drugs residing within the blood trapped in the network of capillaries running throughout brain. Microdialysis can also be used to sample multiple sites within a single animal/person [23, 24]. However, most microdialysis experiments were performed in normal animals rather than in actual animal models of the target disease; this is important because disease status may influence drug disposition in the brain [25–27]. In particular, the BBB is thought to be leaky in individuals with neurodegenerative diseases such as AD or PD [28–31], which has a marked effect on drug passage into the brain.

There are several animal models of PD, such as 6-hydroxydopamine (6-OHDA), rotenone, drosophila *α*-synuclein overexpression, mouse *α*-synuclein overexpression, and 1-methyl-4-phenyl-1,2,3,6-tetrahydropyridine (MPTP) model [32]. 6-OHDA and MPTP are the two classic toxin-induced animal models of PD [33]. MPTP is mainly used in nonhuman primates and in mice [33, 34]. In contrast to primates, rodents are less sensitive to MPTP toxicity [35]. Rats are resistant to MPTP toxicity and mouse strains vary widely in the sensitivity to the toxin [36]. However, 6-OHDA is a highly effective toxin for dopaminergic neurons in mice, rats, cats, and primates [32]. 6-OHDA induced lesion is commonly used in rats, which is established by stereotactic techniques and with relatively low maintenance costs.

A previous investigation in normal animals also indicated that FLZ may be a substrate for the multidrug resistance transporter, P-glycoprotein (P-gp) [17], which is encoded by the* ABCB1 *gene in humans. However, this study only investigated brain-to-plasma ratios at four independent time points after the administration of FLZ; therefore, the time-dependent effect of P-gp inhibition on the distribution of unbound FLZ in the brain was not determined. Furthermore, P-gp function [31, 37] and mRNA expression levels [38] are reportedly reduced in PD brains versus normal brains.

Zosuquidar*·*3HCl is an extremely potent P-gp modulator, does not modulate multidrug resistance protein (MRP1) or breast cancer resistance protein (BCRP) mediated resistance [39, 40], and has a significantly lower affinity for CYP3A than for P-gp [41]. As a third generation P-gp inhibitor, it displays characteristics that make it an “ideal modulator” of P-gp mediated multidrug resistance [42]: it binds P-gp with high affinity; it shows highly potent* in vitro* reversal of drug resistance; it has a high therapeutic index (active at doses ranging from 1 to 30 mg/kg in* in vivo* antitumor efficacy experiments); and it has little effect on the pharmacokinetics of coadministered agents [43–45].

The present study used an innovative* in vivo* microdialysis technique to investigate the penetration of unbound FLZ through the BBB. We also used normal and 6-OHDA induced PD model rats to assess the effects of zosuquidar*·*3HCl (a specific P-gp inhibitor) mediated P-gp inhibition on free FLZ concentrations in the brain ECF and blood over time.* In vivo* microdialysis is a useful tool for evaluating drug passage across the BBB, particularly when used to study drug transporters in the CNS [20]. As such, the present study addresses a knowledge gap regarding the permeability of the BBB to drugs and the effects of P-gp in normal and PD model animals.

## 2. Materials and Methods

### 2.1. Chemicals and Reagents

FLZ (purity = 99.6%; pKa = 13.75 ± 0.46; lipophilic and poorly water-soluble) was supplied by Professor Ping Xie (Institute of Materia Medica, Chinese Academy of Medical Sciences, Beijing, China). Carbamazepine (used as an internal standard (IS)) was obtained from the National Institutes for Food and Drug Control (Beijing, China). Zosuquidar*·*3HCl was purchased from Shanghai Haoyuan Chemexpress Co., Ltd. (Shanghai, China). Bovine serum albumin (BSA, Australian origin; purity > 98%) was purchased from Beijing SeaskyBio Technology Co., Ltd. (Beijing, China). Heparin sodium (purity ≥ 99%, 150 U/mg) was obtained from Beijing Biodee Biotechnology Co., Ltd. (Beijing, China). Acetonitrile (mass spectrometry (MS) grade) was obtained from Honeywell Burdick & Jackson Inc. (Muskegon, MI, USA). Ringer's solution (145 mmol NaCl, 2.97 mmol CaCl_2_, and 4.03 mmol KCl) was purchased from Beijing Double-Crane Pharmaceutical Co., Ltd. (Beijing, China). All other chemicals were high-performance liquid chromatography (HPLC) grade. Deionized water was purified using a Millipore water purification system (Millipore, Billerica, MA, USA). The perfusion fluid was prepared by dissolving BSA in Ringer's solution (4%, w/v).

### 2.2. Experimental Animals

Male Wistar rats (250–350 g; Vital River Laboratories, China) were used in this study. Animals were maintained under temperature-controlled conditions with a 12 h light/dark cycle and allowed food and water* ad libitum*. All rats were allowed to acclimatize for a minimum of 3 days upon arrival before experimentation. All protocols and procedures involving animals were approved by the Animal Care and Welfare Committee of Institute of Materia Medica, Chinese Academy of Medical Sciences and Peking Union Medical College (Beijing, China).

### 2.3. 6-OHDA Induced Rat Model of PD

PD was induced in rats using 6-OHDA, as previously described [46]. Briefly, rats were anaesthetized with pentobarbital sodium (30 mg/kg, i.p.) and mounted on a stereotaxic apparatus. The skull was cleaned and a burr hole was drilled to allow a needle to be passed into the right substantia nigra pars compacta (anterior-posterior: −5.2; lateral: +2.2; dorsal-ventral: −7.8 relative to bregma) according to Paxinos and Watson [47]. Unilateral infusion of 6-OHDA (10 *μ*g dissolved in 0.1% ascorbic acid (final concentration: 2 *μ*g/*μ*L); purity > 98%; Sigma Aldrich, China) was performed for 5 min at a rate of 1 *μ*L/min. After the infusion, the needle was kept in place for another 10 min to prevent leakage along the needle track. To confirm the onset of PD, the rotational behaviour of the rats, which was induced by i.p. injection of apomorphine (0.5 mg/kg, dissolved in water), was tested 14 days after lesion formation. The number of contralateral rotational turns was recorded over 30 min. Immunohistochemical staining for tyrosine hydroxylase (TH) was also performed to confirm PD [6, 48].

### 2.4. Experimental Design

Rats were divided into four groups (*n* = 3 animals per group): three normal rats treated with FLZ only (FLZ only-N); three PD rats treated with FLZ only (FLZ only-PD); three normal rats treated with FLZ plus zosuquidar*·*3HCl (FLZ + ZOSUQ-N); and three PD rats treated with FLZ plus zosuquidar*·*3HCl (FLZ + ZOSUQ-PD). The system was equilibrated for 1 h after surgery. FLZ was dissolved in a mixture of dimethyl sulfoxide, polyethylene glycol 400, and sodium chloride injection (1 : 4 : 5 (v/v/v)). All rats received FLZ (35 mg/kg) via intravenous injection into the tail vein (with or without pretreatment with zosuquidar*·*3HCl). Rats in the FLZ + ZOSUQ-N and FLZ + ZOSUQ-PD groups received an i.v. injection of zosuquidar*·*3HCl (20 mg/kg, dissolved in 20% ethanol-saline) 10 min before FLZ administration. The dose, route of administration, and timing of P-gp inhibition were based on those described in earlier studies [17, 49–51]. Microdialysis samples were collected from the striatum and the jugular vein at 15 min intervals for 1 h before (blanks) and for 4 h after FLZ administration. The samples were stored at −80°C until they were analysed by ultraperformance liquid chromatography-tandem mass spectrometry (UPLC-MS/MS). At the end of the experiment, the rats were decapitated and the brains were removed for histological verification of probe placement. Data were discarded if the probe placement was outside the target area.

### 2.5. *In Vitro* Experiments

*In vitro* experiments were performed to predict nonspecific binding and described approaches to reduce the degree of adsorption based on a previous study [52]. Firstly, the nonspecific binding to the tubing was assessed by comparing the FLZ concentrations before and after passing through the tubing. Then different concentrations of BSA (0.5%, 2%, or 4%, w/v) were added to the perfusion fluid to evaluate the nonspecific binding. The most frequently used calibration method, retrodialysis, was used to assess the level of probe recovery. The general requirement of this method is that the extraction fraction should be the same whether solute exchange across the membrane occurs by either gain (sampling) or loss (delivery) [22]. When the gain value was determined, the probe was inserted into the FLZ standard solution and perfused with the blank perfusion fluid, while the loss value was tested and the probe was positioned in blank perfusion fluid and perfused with FLZ standard solution (gain  = *C*_out_/*C*_*s*_ and loss = 1 − *C*_out_/*C*_in_, where *C*_in_ is the perfusate concentration (inflow to probe), *C*_out_ is the dialysate concentration (outflow from probe), and *C*_*s*_ is the standard FLZ concentration surrounding the membrane). Firstly, BSA was added to the perfusion fluid inside the membrane but not to the solution outside the membrane. Next, BSA (0.5% or 4%) was added to both inside and outside the membrane, respectively. The microdialysate samples were collected every 15 min after an hour of equilibration period.

### 2.6. *In Vivo* Microdialysis Experiments

#### 2.6.1. Microdialysis Surgery

The microdialysis system comprised a CMA 470 refrigerated fraction collector, a CMA 402 syringe pump, and fluorinated ethylene propylene tubing (CMA, Solna, Sweden). CMA microdialysis probes for the blood (CMA 20, 10 mm in length) and brain (CMA 12, 4 mm in length) were also used. Animals were anaesthetized with urethane (1.4 g/kg, i.p.) before surgery and remained anesthetized throughout the experimental period. The body temperature of each rat was maintained at 37°C until the end of the experiment using a heating pad. The blood microdialysis probe was positioned within the jugular vein toward the right atrium and sutured to the surrounding muscle to prevent it from slipping out. The probe was then perfused with heparinized ringer's solution (1500 U/mL) for 15 min. The microdialysis guide cannula was implanted into the right striatum zone (anterior-posterior: 0.2 mm; lateral: 3.0 mm; dorsal-ventral: 3.5 mm from bregma) and fixed to the skull with stainless steel screws and dental acrylic. The brain microdialysis probe was inserted through the guide cannula.

In the pilot microdialysis experiment, ringer's solution was perfused through the probe at a flow rate of 1.5 *μ*L/min and the dialysate samples were collected every 15 min from 90 min before until 6 h after dosing with FLZ. In all the other groups, the probes were perfused with ringer's solution containing 4% BSA, with the flow rate set at 1.0 *μ*L/min.

#### 2.6.2. *In Vivo* Recovery

The relative recovery of FLZ using the microdialysis probe was estimated using a retrodialysis method after sample collection and clearing overnight until there is no drug in the tissue and blood. Perfusates containing FLZ (20 ng/mL for the brain and 200 ng/mL for the blood) were passed separately into the brain and blood through the microdialysis probes at a constant flow rate of 1 *μ*L/min. After a 1-hour stabilization period, the dialysates were collected at 15 min intervals for 1 hour. The concentrations of FLZ in the dialysate (*C*_out_) and perfusate (*C*_in_) were analysed by UPLC-MS/MS as described in Section 2.7. Recovery (*R*) was expressed by the following equation: *R* = 1 − (*C*_out_/*C*_in_).

### 2.7. UPLC-MS/MS Quantification

#### 2.7.1. Preparation of Blood and Brain Samples

The blood and brain samples were prepared by spiking 75 *μ*L of the corresponding IS working solutions (carbamazepine, 10 ng/mL) into 15 *μ*L of dialysate followed by vortex mixing. After centrifuging at 16654 ×g for 10 min, 5 *μ*L of supernatant was injected to the UPLC-MS/MS system.

#### 2.7.2. Instrumentation and Chromatographic Conditions

Sample analysis was performed using a Waters ACQUITY UPLC system (Waters Corp., Milford, MA, USA), and a Waters Xevo triple quadrupole tandem mass spectrometer (Waters Corp., Manchester, UK) was used to detect the analytes. The analytes were separated on a BEH (bridged ethyl hybrid) C_18_ analytical column (50 mm × 2.1 mm, 1.7 *μ*m, Waters Co.) using isocratic elution with a mobile phase consisting of acetonitrile and water containing 0.3% acetic acid (28 : 72, v/v) at a flow rate of 0.45 mL/min. The separation was completed within 3.2 min. After each injection, the needle was washed with acetonitrile for 10 seconds to reduce carryover (a strong wash with 90% acetonitrile followed by a weak wash with 10% acetonitrile). The column was maintained at 30°C and samples were kept at 10°C in the autosampler.

The ESI instrument settings were optimized for the analysis, and the appropriate MRM transitions and MS/MS parameters were determined for individual compounds by direct infusion into the mass spectrometer. The optimum operating parameters of the ESI interface in positive mode were as follows: nebulizer, 7.0 bar; gas flow, 900 L/h; desolvation temperature, 500°C; capillary voltage, 3.5 kV; cone voltage, 40 V; and the LC eluent flow during the period from 0.0 to 1.0 min was switched to waste before introduction to the mass spectrometer for data acquisition. The following precursor-to-product ion transitions were subjected to multiple reaction monitoring: *m*/*z* 450.17→137.03 (cone voltage, 46 V; collision energy, 30 V) and *m*/*z* 450.17→313.08 (cone voltage, 46 V; collision energy, 16 V) as the quantitative ion pair and qualitative ion pair, respectively, for FLZ and 237.07→178.97 (cone voltage, 38 V; collision energy, 32 V) as the quantitative ion pair for carbamazepine (IS). Data acquisition and processing were performed using a Masslynx 4.1 workstation (Waters Corp).

#### 2.7.3. Method Validation

Stock solutions of FLZ and IS were prepared separately in methanol at a target concentration of 1 mg/mL and then diluted with methanol to create working solutions of FLZ at concentrations of 1, 3, 10, 30, 100, 300, 1000, 1500, 2500, 5000, 10000, and 15000 ng/mL and IS at concentrations of 10 ng/mL. All solutions were stored in glass tubes at 4°C for less than 2 months until analysis and were protected from light. FLZ calibration standards (0.1, 0.3, 1, 3, 10, 30, 100, 150, 250, 500, 1000, and 1500 ng/mL) were prepared by spiking the blank perfusate with the appropriate working standard solution of FLZ. Quality control samples were prepared using the working solutions from the same stock solution (0.1, 0.3, 1, 10, 100, and 1000 ng/mL), thereby representing the entire range of concentrations.

Assay validation to meet the acceptance criteria was performed according to the bioanalytical method validation guideline (European Medicines Agency Guideline on Bioanalytical Method Validation, July 21, 2011). The assay was validated in terms of specificity, matrix effect, recovery, calibration curve, precision, accuracy (intra- and interday), lower limit of quantification (LLOQ), and stability (autosampler, freeze-thaw, and long-term).

### 2.8. Data Analysis and Statistical Procedures

Pharmacokinetic parameters were calculated from the observed data by noncompartmental analysis using the Drug and Statistics for Windows software package (DAS, version 2.0, Chinese Pharmacological Association, China). The penetration ratio of FLZ through the BBB was represented by the partition coefficient (Ri), which was calculated as the FLZ area under the concentration-time curve (AUC) in the brain divided by the FLZ AUC in the blood (Ri  = AUC_brain_/AUC_blood_) [53]. A two-sided Student's *t*-test was used to examine differences between the FLZ only-N and FLZ only-PD groups, the FLZ + ZOSUQ-N and the FLZ + ZOSUQ-PD groups, the FLZ only-N and FLZ + ZOSUQ-N groups, and the FLZ only-PD and FLZ + ZOSUQ-PD groups. All data were expressed as the mean ± the standard deviation (SD). The criterion for statistical significance was set at *P* < 0.05.

## 3. Results 

### 3.1. 6-OHDA Induced Rat Model of PD

**Figure 2:**
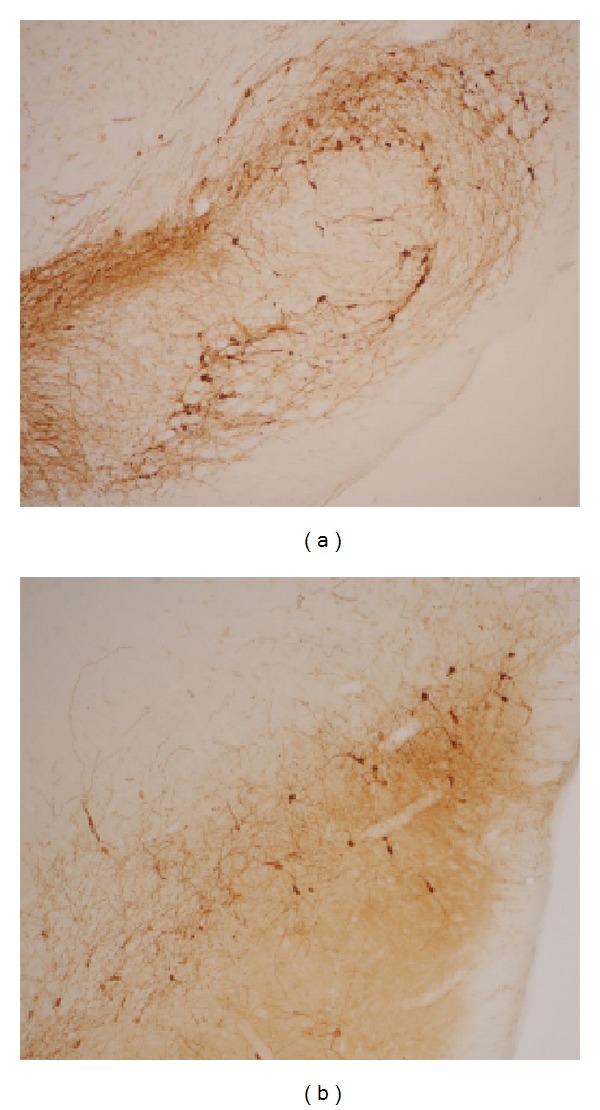
Immunohistochemistry of tyrosine hydroxyls staining. (a) Dopaminergic neurons in normal rat. (b) Dopaminergic neurons in rat with 6-OHDA lesions.

The number of contralateral rotational turns was recorded over 30 min. The rats that rotate more than 210 rotational turns were chosen as the PD rats. Immunohistochemical staining for tyrosine hydroxylase (TH) results showed that the dopaminergic neurons were decreased substantially in the PD rats more than the normal rats (Figure 2).

### 3.2. Method Validation

An accurate, fast, and sensitive UPLC-MS/MS method was developed and validated for the quantification of FLZ in rat brain and blood dialysates. Sample preparation involved “protein precipitation with methanol.” The separation was achieved in 3.2 min, and FLZ and IS were eluted at 2.63 min and 1.55 min, respectively. The LLOQ was 0.1 ng/mL, with no interference by the blank matrix. Two calibration curves (0.1–150 ng/mL and 150–1500 ng/mL; *r* > 0.99) were established to allow the quantification of a wide range of FLZ concentrations. The intraday and interday accuracy were both between 91.7% and 106%, with a precision (represented by the relative standard deviation (RSD)) < 11%. The mean extract recovery was >73.2%. The matrix effect was between 1.04 and 1.10, with an RSD < 13%. The stability of FLZ during preparation, after three freeze/thaw cycles and after storing at −80°C for 45 days, was also monitored. The results indicated that FLZ remained stable under all three conditions.

### 3.3. *In Vitro* Experiments

More than 90% of FLZ was bound to the tubing when perfused with ringer's solution containing FLZ. This value was less than 15% after the addition of BSA (0.5%, 2%, or 4%, w/v) to the perfusion fluid. The gain value increased substantially as the BSA concentration in the perfusion fluids increased; however, the loss value changed in the opposite direction. The two values were significantly different. The gain and loss values were very close when the BSA content was added up to 4% to both sides of the membrane. Application of these new perfusion conditions resulted in a marked increase in the relative* in vivo *recovery of FLZ (13.2% and 29.0%, resp., for the brain and blood microdialysis probes). Furthermore, these new conditions were similar to those encountered in actual* in vivo* systems, in which albumin is present in both the plasma and ECF.

### 3.4. Free FLZ Pharmacokinetics

Because the FLZ levels in the brain dialysate were below the LLOQ after administration of FLZ for 3.5 hours, samples were collected from the FLZ only-N and FLZ only-PD groups during this period only; the mean* in vivo* recovery by the blood and brain probes was 29.0 ± 2.1% and 13.2 ± 3.5%, respectively. The FLZ concentrations in the dialysate were calibrated by the recovery values as the following equation: *C* = *C*_*d*_/*R* (where *C* is the actual free FLZ concentration in the ECF or blood, *C*_*d*_ is the determined free FLZ concentration in the dialysate, and *R* is the FLZ recovery by the probe).

**Figure 3:**
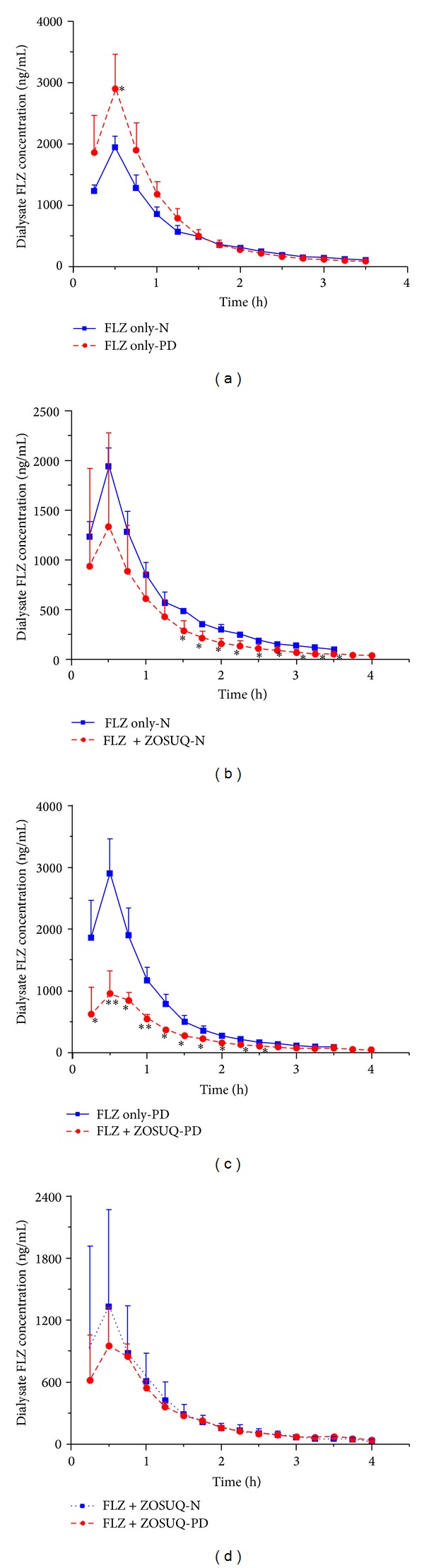
Blood dialysate FLZ profiles for the different treatment groups (*n* = 3 per group). (a) FLZ concentrations measured in blood dialysates at different time points in the FLZ only-N and FLZ only-PD groups. A significant difference was observed at the second time point only (30 min). (b) FLZ concentrations measured at different time points in blood dialysates from the FLZ only-N and FLZ + ZOSUQ-N groups. FLZ levels decreased significantly between the sixth and fourteenth samples in the FLZ + ZOSUQ-N group relative to those in the FLZ only-N group. (c) FLZ concentrations measured at different time points in blood dialysates from the FLZ only-PD and FLZ + ZOSUQ-PD groups. There was a significant reduction in FLZ levels in the FLZ + ZOSUQ-PD compared with the FLZ only-PD group (the first to the tenth sample). (d) FLZ concentrations in blood dialysates measured at different time points in the FLZ + ZOSUQ-N and FLZ + ZOSUQ-PD groups. There was no statistical difference in FLZ levels between the two groups. *P < 0.05; **P < 0.01. Data are expressed as the mean ± SD. Note: FLZ dialysate samples from the FLZ only-N and FLZ only-PD groups were collected for 3.5 hours (brain dialysate concentrations after this period were below the limit of quantification).

**Figure 4:**
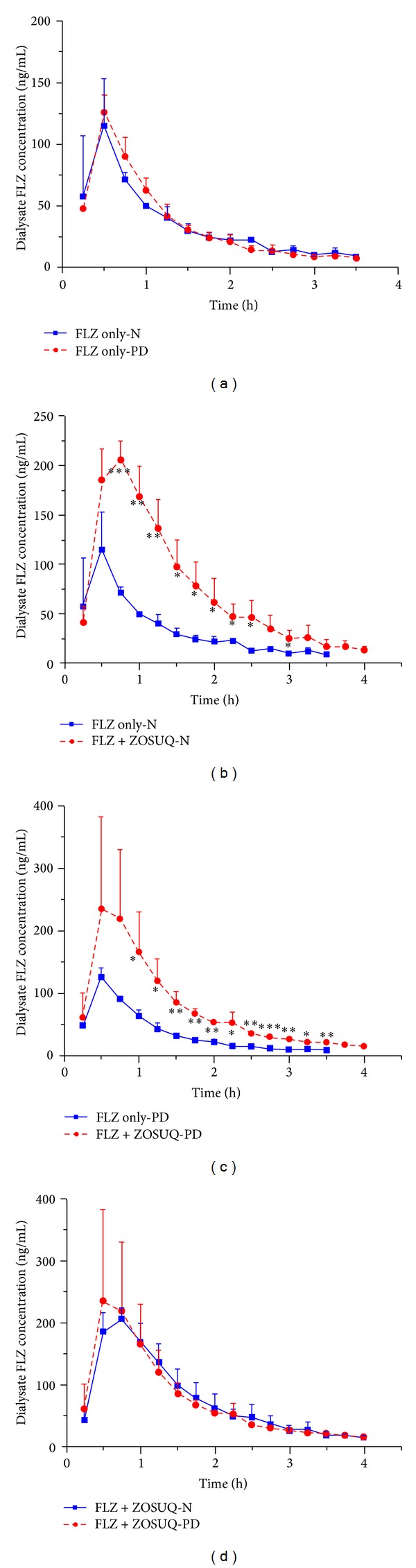
Brain dialysate FLZ profiles in the different groups (*n* = 3 per group). (a) FLZ concentrations in brain dialysate samples collected at different times from the FLZ only-N and FLZ only-PD groups. The differences in FLZ levels between these two groups were not significant. (b) FLZ concentrations in brain dialysate samples taken at different times from the FLZ only-N and FLZ + ZOSUQ-N groups. FLZ concentrations were significantly higher in the FLZ + ZOSUQ-N group than in the FLZ only-N group (between the third and twelfth samples; the eleventh sample is the exception). (c) FLZ concentrations in brain dialysate samples taken at different times from the FLZ only-PD and FLZ + ZOSUQ-PD groups. FLZ concentrations were significantly higher in the FLZ + ZOSUQ-PD group than in the FLZ only-PD group (between the fourth and fourteenth samples). (d) FLZ concentrations in brain dialysate samples taken at different times from the FLZ + ZOSUQ-N and FLZ + ZOSUQ-PD groups. There was no statistically significant difference in FLZ levels between these two groups. *P < 0.05; **P < 0.01; ***P < 0.001. Data are expressed as the mean ± SD. Note: FLZ dialysate samples were collected from the FLZ only-N and FLZ only-PD groups for 3.5 hours (brain dialysate concentrations after this period were below the limit of quantification).

Statistical analysis indicated that there was no significant difference between the FLZ only-N and FLZ only-PD groups in terms of free FLZ levels in the blood and brain dialysates over time (*P* > 0.05, Figures 3(a) and 4(a)) (the only exception was the second blood dialysate sample; *P* < 0.05). In addition, there were no significant differences between the two groups in terms of pharmacokinetic parameters (AUC; half-life of drug (*t*_1/2_); drug clearance from plasma (Cl); Table 1). Similarly, there were no significant differences in FLZ levels or pharmacokinetic parameters between the FLZ + ZOSUQ-N and FLZ + ZOSUQ-PD groups (*P* > 0.05, Figures 3(d) and 4(d) and Table 1).

Nonetheless, FLZ levels in the FLZ only-N and FLZ + ZOSUQ-N groups showed a significant group effect over time. Blood dialysate FLZ concentrations were significantly higher in the FLZ only-N group than in the FLZ + ZOSUQ-N (*P* < 0.05, Figure 3(b)) group between the sixth and fourteenth samples (inclusive), and FLZ concentrations in the brain dialysates from the FLZ + ZOSUQ-N group were significantly higher than in those from the FLZ only-N group between the third to the twelfth samples (the only exception was the eleventh sample) (*P* < 0.05, Figure 4(b)). Correspondingly, the mean brain dialysate FLZ AUC was significantly higher for the FLZ + ZOSUQ-N group than for the FLZ only-N group (123% increase; *P* < 0.01, Table 1); however, the mean difference in the blood dialysate FLZ AUC between the two groups was not significant (*P* > 0.05, Table 1).

Statistically significant differences were also observed between the FLZ only-PD group and FLZ + ZOSUQ-PD group. The FLZ concentrations in the blood dialysates (from the first to the tenth samples) from the FLZ + ZOSUQ-PD group were significantly lower than those from the FLZ only-PD group (*P* < 0.05, Figure 3(c)), whereas the FLZ concentrations in the brain dialysate were significantly higher between the fourth and the fourteenth samples (*P* < 0.05, Figure 4(c)). The decrease in the FLZ blood dialysate AUC (56% decrease; *P* < 0.05, Table 1) and the increase in the FLZ brain dialysate AUC (130% increase; *P* < 0.05, Table 1) observed in the FLZ + ZOSUQ-PD group relative to the FLZ only-PD group were statistically significant.

### 3.5. Comparison of Brain to Blood Free FLZ AUC Ratios

**Figure 5:**
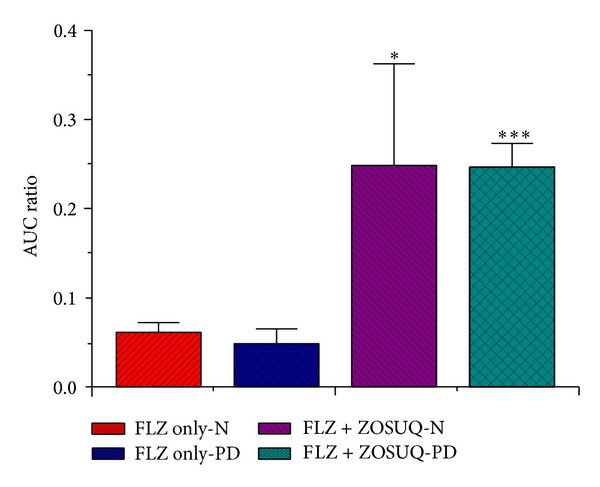
Brain dialysate : blood dialysate FLZ AUC ratios for each treatment group. There was a 3-fold increase in the brain dialysate : blood dialysate FLZ AUC ratio in the FLZ + ZOSUQ-N group relative to that in the FLZ only-N group (*P* < 0.05) and a 4-fold increase in the FLZ + ZOSUQ-PD group relative to that in the FLZ only-PD group (*P* < 0.001). The 20% reduction observed in the FLZ only-PD group relative to the FLZ only-N group was not statistically significant (*P* > 0.05). The mean ratios in the FLZ + ZOSUQ-N and FLZ + ZOSUQ-PD groups were not significantly different (*P* > 0.05). Data are expressed as the means ± SD (*n* = 3 per group).

The dialysate brain : blood AUC ratios give an indication of BBB transport as they account for variations in FLZ levels, which might explain any observed differences in dialysate concentrations. Statistical analysis revealed that the 3-fold increase in the brain : blood free FLZ AUC ratio observed in the FLZ + ZOSUQ-N group relative to the FLZ only-N group was statistically significant (*P* < 0.05, Figure 5) and that the 4-fold increase in the brain : blood free FLZ AUC ratio observed in the FLZ + ZOSUQ-PD group relative to the FLZ only-PD group was also statistically significant (*P* < 0.001, Figure 5). The 20% decrease in the blood : brain free FLZ AUC ratio observed in the FLZ only-PD group relative to the FLZ only-N group was not statistically significant (*P* > 0.05, Figure 5). Also, the ratios in the FLZ + ZOSUQ-N and FLZ + ZOSUQ-PD groups were not significantly different (*P* > 0.05, Figure 5).

## 4. Discussion

A pilot* in vivo* experiment was performed to obtain basic information about the feasibility of using microdialysis to assess unbound FLZ levels in the brain and blood of normal rats. The levels of FLZ in brain and blood dialysate samples were very low in most of the postdose samples with no defined time course for FLZ (data not shown); also, the* in vitro* recovery was less than 1%. This may be explained by the very low recovery of FLZ by the microdialysis probes and/or by the adsorption of the compound to the outflow tubing or to the microdialysis probes themselves [54].

Subsequent* in vivo* microdialysis experiments were performed in rats dosed with FLZ (35 mg/kg, i.v.) under the modified perfusion conditions. There was no significant difference in FLZ BBB penetration or pharmacokinetic parameters in the blood or brain between the FLZ only-N and FLZ only-PD groups, which differs from the previous studies reporting alterations/damage in blood brain barrier after 6-OHDA administration [48, 55] or in PD patients [30, 31]. It may be because of the fact that the 6-OHDA induced rat model was an acute brain injury model which may lead to BBB dysfunction on one side, while increasing P-gp expression on the other side [55]. FLZ was a substrate of P-gp at the same time, which would affect the FLZ penetration to BBB. The brain dialysate FLZ AUC for animals in the FLZ + ZOSUQ-N group was 123% higher than that for animals in the FLZ only-N group, and that in the FLZ + ZOSUQ-PD group was 130% higher than that in the FLZ only-PD group. These observed differences were strongly suggestive of increased FLZ transport across the BBB in zosuquidar*·*3HCl-treated animals. The mean free FLZ AUC in the blood dialysates from the FLZ + ZOSUQ-PD group was 56% lower than that for animals in the FLZ only-PD group significantly. There was also a 34% reduction in the FLZ AUC for the FLZ + ZOSUQ-N group compared with the FLZ only-N group, although the difference was not statistically significant, possibly due to the increased variability in FLZ + ZOSUQ-N group. Pretreatment with zosuquidar*·*3HCl caused a reduction in the FLZ AUC for the blood dialysate; this may be because more FLZ penetrated the BBB and became distributed throughout the brain (or other tissues that express P-gp at high levels). The brain to blood free FLZ AUC ratio for animals in the FLZ + ZOSUQ-N group was 3 times higher than that for animals in the FLZ only-N group, and the ratio for animals in the FLZ + ZOSUQ-PD group was 4 times higher than that for animals in the FLZ only-PD group. Our previous study [17] showed that, compared with that in the control group, the FLZ brain-to-plasma ratio in animals pretreated with zosuquidar*·*3HCl increased by about 3.1-fold at 0.25 min postdose and by about 14.5-fold to 20.8-fold at the other three time points tested (4, 10, and 30 min postdose). The increases noted in the present study were lower than that. This may be due to the different sampling methods used (the previous study used trunk blood and brain homogenate collected following decapitation) or different data analysis methods (the previous study used the brain homogenate-to-plasma concentration ratio rather than the brain dialysate AUC-to-blood dialysate AUC ratio). There was no significant difference in FLZ BBB penetration between the FLZ + ZOSUQ-N and the FLZ + ZOSUQ-PD groups or between the FLZ only-N and FLZ only-PD groups, further supporting the hypothesis that P-gp limits the ability of FLZ to penetrate the BBB [17]. In addition, the *t*_1/2_ of FLZ in the present study was about 1.1 h (both in the blood and brain), which is longer than that reported previously (about 0.5 h) [17]. This may be due to the high level of FLZ binding to plasma proteins. This result also indicates that it is important to ascertain free drug levels when undertaking pharmacokinetic studies of CNS drugs that bind tightly to plasma proteins.

To the best of our knowledge, this is the first study to examine the effects of a P-gp modulator (zosuquidar*·*3HCl) on the transport of free FLZ across the BBB. The present study was also the first to use the* in vivo* microdialysis technique to investigate the pharmacokinetic characteristics of FLZ in the rat brain. However, the present study still has limitations. Firstly, the 6-OHDA model, as an acute animal model, differs from the slowly progressive pathology of human PD and does not mimic all pathological and clinical features of human Parkinsonism, although it was frequently used in the preclinical studies. We should note the differences between PD model animals and PD patients in future clinical trial of FLZ. Besides, the animals were anesthetized during sampling, which may have altered the pharmacokinetics of the drug. Future studies should use animals that are conscious and able to move freely, and the levels of FLZ and the relative neurotransmitters (such as dopamine and its metabolites) should be determined at the same time to establish a relationship between pharmacokinetics and pharmacodynamics under the normal and disease states. Such studies may help us to identify the pharmacological mechanism of action.

In conclusion, the present study suggests that BBB penetration by FLZ is similar in normal and PD rats. The bioavailability of FLZ in the brains of rats pretreated with zosuquidar*·*3HCl increased in both normal and PD rats, suggesting that P-gp efflux limits the ability of FLZ to cross the BBB. These findings suggest that P-gp prevents FLZ from reaching its target site in the brain. The approach described herein might be useful for investigating BBB penetration by other drugs used to treat CNS disease.

## Figures and Tables

**Table 1:** Key pharmacokinetic parameters and FLZ BBB penetration values (*n* = 3 per group).

		FLZ only-N	FLZ only-PD	FLZ + ZOSUQ-N	FLZ + ZOSUQ-PD
AUC (*μ*g/L × h)	Blood	2349 ± 39	3072 ± 621	1562 ± 881	1334 ± 259∗
	Brain	145 ± 25	145 ± 18	324 ± 46∗∗	333 ± 103∗
*t*_1/2_ (h)	Blood	1.21 ± 0.22	1.34 ± 0.03	1.11 ± 0.13	1.43 ± 0.25
	Brain	1.15 ± 0.57	1.31 ± 0.29	1.04 ± 0.43	1.14 ± 0.27
CL_z_ (L/h/kg)	Blood	14.9 ± 0.2	11.7 ± 2.5	28.4 ± 16.8	26.8 ± 4.7∗∗
Ri (AUC_brain_/AUC_blood_)		0.0617 ± 0.0105	0.0492 ± 0.0158	0.2485 ± 0.1142∗	0.2459 ± 0.0270∗∗∗

*P < 0.05; **P < 0.01; ***P < 0.001.

## Abbreviations

AD: Alzheimer's disease
AUC: Area under the concentration-time curve
BBB: Blood-brain barrier
BEH: Bridged ethyl hybrid
BSA: Bovine serum albumin
*C*_in_: Concentration of FLZ in the perfusate
Cl: Drug clearance from plasma
*C*_out_: Concentration of FLZ in the dialysate
CNS: Central nervous system
COMT: Catechol-O-methyltransferase
CSF: Cerebrospinal fluid
ECF: Extracellular fluid
FLZ: N-[2-(4-Hydroxy-phenyl)-ethyl]-2-(2,5-dimethoxy-phenyl)-3-(3-methoxy-4-hydroxy-phenyl)-acrylamide
FLZ only-N group: FLZ only in normal rats
FLZ only-PD group: FLZ only in PD rats
FLZ + ZOSUQ-N group: FLZ plus zosuquidar*·*3HCl in normal rats
FLZ + ZOSUQ-PD group: FLZ plus zosuquidar*·*3HCl in PD rats
HPLC: High-performance liquid chromatography
IS: Internal standard
L-DOPA: 3,4-Dihydroxy-L-phenylalanine
LLOQ: Lower limit of quantification
MAO-B: Monoamine oxidase-B
MPTP: 1-Methyl-4-phenyl-1,2,3,6-tetrahydropyridine
MRP: Multidrug resistance protein
MS: Mass spectrometry
NSB: Nonspecific binding
P-gp: P-Glycoprotein
*t*_1/2_: Half-life of drug
PD: Parkinson's disease
RSD: Relative standard deviation
SD: Standard deviation
TH: Tyrosine hydroxylase
UPLC-MS/MS: Ultraperformance liquid chromatography-tandem mass spectrometry
Zosuquidar*·*3HCl: Zosuquidar trihydrochloride
6-OHDA: 6-Hydroxydopamine.
